# Raising awareness for cardiovascular autonomic dysfunction: the 2023 European Society of Hypertension guidelines revisited

**DOI:** 10.1007/s10286-023-00980-8

**Published:** 2023-09-19

**Authors:** Jens Jordan, Italo Biaggioni

**Affiliations:** 1 Institute of Aerospace Medicine, German Aerospace Center (DLR), Linder Hoehe, 51147 Cologne, Germany; 2 Medical Faculty, University of Cologne, Cologne, Germany; 3 Autonomic Dysfunction Center and Division of Clinical Pharmacology, Department of Medicine, Vanderbilt University Medical Center, Nashville, TN USA

**Keywords:** Hypertension, Blood pressure, Autonomic disorders, Guidelines, Baroreflex failure, Autonomic failure

The clinical guidelines of the European Society of Hypertension (ESH) are a resource used not only by hypertension specialists but also by general physicians, internists, and other medical specialties. While the focus remains on management of hypertensive disorders, for the first time, the new 2023 guidelines include recommendations for diagnosis and management of autonomic disorders, including autonomic failure and (afferent) baroreflex failure among others [1]. This is a welcomed addition and one that will raise awareness of cardiovascular autonomic diseases among non-autonomic specialists. In this editorial, we will give an overview on the areas that are covered by the new guidelines, relevant to patients with autonomic disorders.

Because blood pressure and heart rate are tightly regulated through baroreflex adjustments of sympathetic and parasympathetic efferent nerves, patients with autonomic disorders often present with cardiovascular signs and symptoms. The most obvious consequence of autonomic cardiovascular disease is an inability to regulate blood pressure with standing, which makes orthostatic blood pressure and heart rate measurements a useful clinical screening test. The 2023 ESH guidelines recommend the measurement of orthostatic vitals as a screening tool in persons older than 65 years and in patients with treated arterial hypertension, diabetes mellitus, neurodegenerative disorders, or with orthostatic symptoms [1]. The recommendation is to measure blood pressure after 1 and 3 min standing to detect orthostatic hypotension. The guidelines also suggest that ambulatory or home blood pressure measurements should be considered when orthostatic or postprandial hypotension is suspected [1]. This approach may have the added benefit of diagnosing supine hypertension, which occur in more than half of patients with orthostatic hypotension [2].

The 2023 ESH guideline refers to the American Autonomic Society recommendations to define orthostatic hypotension as a reduction in systolic blood pressure of at least 20 mmHg or in diastolic blood pressure of at least 10 mmHg within 3 min of standing [3]. The ESH guidelines also discuss the opposite phenomenon, the abnormal increase in blood pressure on standing [1]. The authors suggest that there is no generally accepted definition of orthostatic hypertension, but a resent consensus document that was published in this Journal made the distinction between an exaggerated pressor response, defined as a sustained systolic blood pressure increase of at least 20 mmHg when changing from the supine to the standing position, and orthostatic hypertension, when an exaggerated pressor response is associated with upright systolic blood pressure of at least 140 mmHg [4, 5]. Population studies find similar prevalence of both extremes of blood pressure response to standing, orthostatic hypotension, and orthostatic hypertension, and epidemiological studies indicate that both conditions herald increased cardiovascular risk [6–8]. Even less pronounced changes in blood pressure with standing not reaching these diagnostic cutoff levels may be associated with poor outcomes. We believe, therefore, that recognition of orthostatic hypotension and orthostatic hypertension is important for risk stratification.

Autonomic specialists are well aware that the clinical phenotypes of autonomic disorders can vary profoundly depending on the site of the lesion [9]. However, physicians, rarely encountering patients with autonomic diseases, may not be familiar with these differences, and the diagnosis is often delayed or incorrect such that potentially effective therapies are not instituted. An important contribution of the 2023 ESH guideline is that clinical features and management approaches of (afferent) baroreflex failure and (efferent) autonomic failure are clearly distinguished [1]. Despite their completely different presentations, these conditions are often confused and mislabeled even in the medical literature. A nearly complete loss of baroreflex afferent function causes baroreflex failure, which is characterized by volatile arterial hypertension that is exacerbated by psychological and physiological stress. The diagnosis should be considered when these symptoms are accompanied by predisposing conditions such as previous neck dissection or radiation therapy. The guideline also suggests that the diagnosis baroreflex failure should be confirmed by baroreflex testing preferably in specialized centers and that long-acting sympatholytic drugs can be utilized to ameliorate hypertensive surges.

In contrast, parasympathetic and sympathetic efferent dysfunctions cause autonomic failure. The ESH 2023 guidelines suggest that underlying causes should be sought for to identify potentially treatable conditions, such as autoimmune autonomic ganglionopathy, and to gauge prognosis [1]. Moreover, for patients with symptomatic orthostatic hypotension, non-pharmacological treatment options including increased sodium ingestion, water ingestion, and venous compression garments are recommended as first-line therapy. Diuretics, alpha-1 adrenoreceptor blockers, vasodilators, and other medications that can worsen orthostatic hypotension should be discontinued whenever possible. For patients who remain symptomatic, pharmacological therapy of the orthostatic hypotension can be considered. The guideline points out that these treatments can worsen supine hypertension. The ESH 2023 guideline, similarly to a previous consensus document, suggests that in patients with supine hypertension, sleeping with the head of the bed tilted up can be tried to reduce blood pressure [1, 10]. Yet, pharmacotherapy of supine hypertension could be considered in selected patient after individual risk–benefit consideration. In particular, potential benefits of antihypertensive therapy on cardiovascular outcomes should be weighed against risk of fall and overall prognosis of the underlying condition [1].

Overall, the recommendations regarding management of autonomic cardiovascular disease reflect what most experts treating these patients have practiced for years. The importance of such recommendations in a clinical guideline is a broader distribution of knowledge across medical disciplines. One potential benefit is that patients may be diagnosed at an earlier stage and, when required, be transferred to specialist care. However, most of the recommendations for patients with cardiovascular autonomic disease have a relatively low level of evidence. In fact, all recommendations for patients with baroreflex failure or autonomic failure are supported by a level of evidence-C-meaning that the recommendation mainly relies on observational studies, surrogate outcomes, or expert opinion. A potential risk of guidelines is that in clinical practice, recommendations are used as template for clinical decisions without consideration of the strength of the evidence. Clearly, we need better-quality studies in the future to inform clinical decision-making. Perhaps, the ESH 2023 guideline could create an impetus to embark on larger and better designed clinical studies in these patients. Yet, the relatively low number of affected patients poses an important limitation.

A final comment, which may be beyond the scope of this text, is that in most patients with “garden variety” arterial hypertension, the autonomic nervous system contributes to the increase in blood pressure. Even when the autonomic nervous system is not the primary driver of the blood pressure increase, the baroreflex set-point is usually reset to a higher blood pressure level such that autonomic mechanisms help to sustain the condition. Therefore, interventional therapies targeting the sympathetic nervous system are discussed in the ESH 2023 guideline [1]. We propose that the deep knowledge that autonomic scientists and clinicians have gathered over the years could be useful when scrutinizing and targeting these therapies.

## Data Availability

Not applicable.
